# Feasibility of ultrasound-based identification of correct central venous access using two acoustic windows

**DOI:** 10.1186/2197-425X-3-S1-A607

**Published:** 2015-10-01

**Authors:** G Zick, G Elke, D Schaedler, T Becher, L Balke, N Weiler

**Affiliations:** Department of Anesthesiology and Intensive Care Medicine, University Medical Center Schleswig-Holstein, Campus Kiel, Kiel, Germany; Department of Internal Medicine I, University Medical Center Schleswig-Holstein, Campus Kiel, Kiel, Germany

## Introduction

To avoid unnecessary X-ray examinations for confirming the correct placement of a central venous catheter and ruling out complications the following two criteria have to be met: 1) Confirmation of the correct placement in the vena cava, 2) Exclusion of pneumothorax and haemothorax. The latter can be accomplished using ultrasound [1], [2]. To prove the correct placement in the vena cava an ECG based approach has been suggested. Since this is only reliable with sinus rhythm patients with atrial fibrillation pose a problem. The following approach offers a safe alternative method in this population.

## Objectives

The aim of this pilot study was to examine the feasibility of using ultrasound with two acoustic windows to visualize the j-wire in the vena cava.

## Methods

In eight consecutive critically ill patients requiring a central venous catheter the vena cava was visualized from the right body side of the patient through the liver or the subcostal window displaying the heart with the connection of the vena cava to the right atrium. This examination was performed using the vivid S6 with a sector scanner (M4S-RS, 1.5 to 3.6 MHz) or convex scanner (4C-RS, 2 to 5 MHz; GE Healthcare, Munich, Germany). It accompanied the insertion of the j-wire before dilatation and insertion of the catheter.

## Results

The j-wire could be identified in all eight patients from the right body side and in seven out of eight patients with the subcostal approach.

## Conclusions

The visualization of the j-wire in the vena cava is a simple non-invasive bedside ultrasound-based method shown to be reliable in our pilot study. It confirms the correct placement of central venous catheters. Complications like pneumothorax and haemothorax can also be ruled out with ultrasound of the pleura. A radiological X-ray examination would be no longer needed with this indication. As an additional benefit, an unintended arterial placement would be identified before dilatation of the vessel.

## References

[1] Lichtenstein D, van Hooland S, Elbers P (2014). Ten good reasons to practice ultrasound in critical care. Anaesthesiol Intensive Ther.

[2] Lichtenstein DA, Meziere G, Lascols N (2005). Ultrasound diagnosis of occult pneumothorax. Crit Care Med.

